# Adipose Mesenchymal Stem Cell-Derived Exosomes Promote Wound Healing Through the WNT/β-catenin Signaling Pathway in Dermal Fibroblasts

**DOI:** 10.1007/s12015-022-10378-0

**Published:** 2022-04-26

**Authors:** Cong Li, Yu An, Yu Sun, Fan Yang, Quanchen Xu, Zhiguo Wang

**Affiliations:** 1 The Affiliated Hospital of Qingdao University, Qingdao University, Qingdao, Shandong Province, 266021 People’s Republic of China; 2 Department of Stomatology, the Affiliated Hospital of Qingdao University, Qingdao University, Qingdao, Shandong Province, 266021 People’s Republic of China; 3 Department of Burn and Plastic Surgery, the Affiliated Hospital of Qingdao University, Qingdao University, Qingdao, Shandong Province, 266021 People’s Republic of China

**Keywords:** Wound healing, Exosomes, Adipose mesenchymal stem cells, Fibroblasts, WNT/β-catenin signaling pathway

## Abstract

The differentiation, migration, and proliferation of skin fibroblasts are identified as key factors in cutaneous wound healing. Adipose-derived mesenchymal stem cells (ADMSCs) and their exosomes (ADMSC-Exos) have been considered as potential therapeutic tools for tissue regeneration; however, the underlying mechanisms on cutaneous wound healing are still not well understood. In this study, we successfully obtained ADMSC-Exos and found ADMSC-Exos significantly promoted the migration and proliferation of fibroblasts in a dose-dependent manner in vitro. The expression levels of COL-I and COL-III in fibroblasts treated with ADMSC-Exos were significantly increased, while the expression level of α-SMA was decreased. In addition, the enhanced protein expression of WNT2b and β-catenin confirmed the activation of the WNT/β-catenin signaling pathway and the WNT/β-catenin inhibitor (XAV939) reversed the promoting effect of ADMSC-Exos on wound healing and the β-catenin expression. Taken together, our study partially elucidates the mechanism of ADMSC-Exos in wound healing, illustrating the potential of ADMSC-Exos as a new therapeutic approach to promote skin wound healing.

**Graphical Abstract:**

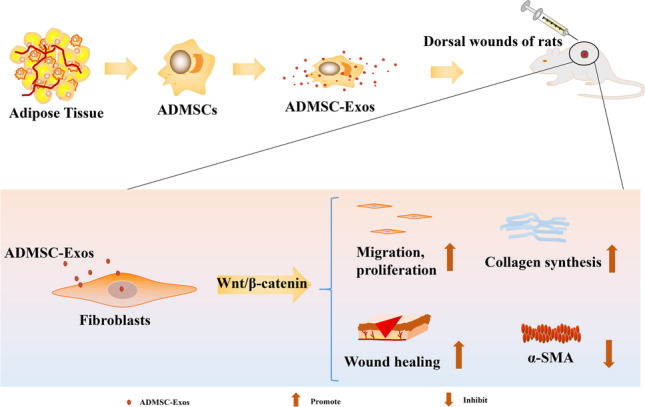

## Introduction

Cutaneous wound healing is a complex and continuous process that includes haemostatic, inflammatory, proliferative, and remodelling stages. Various factors, such as high wound tension, infection, radiation damage, and metabolic disease, lead to prolonged wound healing time, causing physical or mental pain and imposing a severe treatment burden on patients [1, 2]. Fibroblasts are the most abundant cells in the dermis and have the ability to synthesize and remodel the extracellular matrix (ECM). During different wound healing stages, integration of the proliferation, differentiation, and migration of skin fibroblasts plays an important role [3–5]. Therefore, regulation of fibroblasts is a very important target in scar treatment.

Recently, cell therapy has received much attention for its applications in skin tissue repair [6]. Adipose-derived mesenchymal stem cells (ADMSCs) have become one of the most promising stem cell populations identified to date because they can be harvested in larger quantities easily with minimal damage to the donor site [7–9]. Numerous studies have reported that ADMSCs can promote the proliferation and migration of fibroblasts and epidermal cells during tissue injury, promote the formation of new blood vessels during ischaemic tissue injury, and finally accelerate tissue regeneration and wound healing [10–12]. However, as the high clearance rate of stem cells in vivo and their potential carcinogenic risk, the clinical application of stem cells is limited [13]. Recent studies have shown that one of the mechanisms of action of mesenchymal stem cells (MSCs) is mediated through the paracrine pathway, in which the discovery of exosomes is highly important [14, 15].

Exosomes enclosed by lipid membranes are a type of extracellular vesicle secreted by cells that contain a variety of microRNAs, proteins, cytokines, lipids, and unedited RNAs [16]; thus, they have information transmission functions similar to those of the source cell and can transport a variety of cellular components to target cells. Many cells, such as lymphocytes, tumour cells, MSCs, oligodendrocytes, epithelial cells, etc., have been indicated to secrete exosomes [16]. Exosomes are formed in cells via endocytosis [17]. Exosomes play an essential role in regulating different physiological and pathological processes, such as substance transport, cell survival, apoptosis, and cell proliferation. Furthermore, exosomes can regulate angiogenesis and immune regulation and can reduce ischaemia–reperfusion injury [18]. In addition, exosomes have the following characteristics: long-term activity, easy transport, low immunogenicity, easy control of the concentration and modification of their contents with the microenvironment [19]. Researchers believe that exosomes are the paracrine effectors of MSCs, which participate in a wide range of biological processes by affecting tissue responses to injury, infection, and disease [16], while the mechanism is poorly understood [17].

The WNT/β-catenin signaling pathway is a classical WNT signaling pathway that plays an important role in wound healing [20]. Our previous studies showed that WNT-responsive stem cells residing in the oral mucosa and bone are responsible for oral wound healing[21, 22]. There is also evidence that MSCs-derived exosomes may play a role in promoting cardiovascular disease healing by activating the WNT/β-catenin signaling pathway [23]. However, the relationship between ADMSC-Exos and the WNT/β-catenin signaling pathway in wound healing remains unclear.

In this study, we isolated ADMSC-Exos by an ultracentrifugation method and investigated their effect on normal skin fibroblasts to explore their role in the wound healing process. We also used an SD rat model of skin defects to investigate the potential relationship between ADMSC-Exos and the WNT/β-catenin signaling pathway in wound healing. Therefore, this study aimed to clarify the regenerative effects of ADMSC-Exos in cutaneous wound repair.

## Materials and Methods

### Isolation and Characterization of ADMSCs

Human adipose tissue, which was obtained from patients undergoing liposuction at the Affiliated Hospital of Qingdao University, was digested with 0.075% collagenase type I (Invitrogen, USA). The digestion reaction was stopped using complete DMEM (HyClone, China), and the solution was then centrifuged at 1300 × g (Beijing Era Beili, China) for 10 min. After resuspension in medium containing 15% FBS (Procell, China), the ADMSC solution was transferred to culture dishes and cultured at 37 °C in 5% CO_2_. Images of typical areas were acquired with a microscope (Olympus Corporation, Japan).

After passage 3 ADMSCs were prepared into a cell suspension (1 × 10^3^ cells/ml), they were divided into 6 parts and transferred to centrifuge tubes. Then, PE-anti-human CD29 (# FAB17781P; R&D Systems, USA), 488-anti-human CD44 (# FAB3660G; R&D Systems, USA), PE-anti-human CD45 (# FAB1430P-100; R&D Systems, USA), PE-anti-human CD90 (# FAB2067P; R&D Systems, USA), and 488-anti-human CD105 (# FAB10971G-100UG; R&D Systems, USA) antibodies and PBS were added to centrifuge tubes (5 μl each) and incubated at room temperature in the dark for 30 min. After centrifugation at 900 × g for 5 min, the samples resuspended in 200 μl of PBS for flow cytometry. The data were analyzed with FlowJo 7.6.1 software.

### Induction of Osteoblastic and Adipogenic Differentiation of ADMSCs

After passage 3 ADMSCs were grown to a confluence of 80% to 90%, the medium was replaced with osteoblast induction medium (ScienCell, USA) or adipogenic induction medium (ScienCell, USA). Alkaline phosphatase (Beyotime, China) staining was performed on the 9th day of induction, and Alizarin red S (Solarbio, China) staining was performed on the 21st day of induction to evaluate osteogenesis. Oil red O (Solarbio, China) staining was performed on the 14th day of induction to evaluate lipid droplet formation. Staining was observed and imaged under a microscope (Olympus Corporation, Japan).

### Isolation and Characterization of ADMSC-Exos

The exosome isolation procedure was based on our previous study and modified appropriately [24]. In brief, cells were cultured in serum-free medium for 36 h to a confluence of 70% ~ 80%. After collection, the ADMSC media were centrifuged (Beijing Era Beili, China) for 10 min at 300 × g, 10 min at 2000 × g, and 30 min at 10 000 × g to remove dead cells and large pieces of cell debris. The supernatant was filtered through a 0.22 µm-pore syringe filter (Millipore Millex-GP, USA) to remove cell debris and large vesicles. The supernatant was then ultracentrifuged twice at 100 000 × g for 70 min at 4 °C using a 70Ti rotor (Beckman Coulter, USA). The obtained exosomes were resuspended in 200 µl of PBS, stored at -80 °C and used for experiments as soon as possible. The exosome suspension concentration was determined according to the instructions of a BCA protein concentration assay kit (Elabscience, China). Exosome morphology was observed by transmission electron microscopy. The particle diameter distribution was measured by nanoparticle tracking analysis (NKT, China).

### Wound Healing Experiments in Rats

To evaluate the effects of ADMSC-Exos on wound healing, a total of 9 male SD rats (270 g ~ 290 g) (Pengyue Laboratory Animal Breeding Co. Ltd, China) were randomly divided into a blank control group, PBS treatment group and ADMSC-Exos treatment group. Full-thickness round wounds of equal sizes (0.8 × 0.8 cm^2^) were aseptically generated in the middle of the dorsal skin, and a ring-shaped rubber ring was sewn around the wound and left in place for 10 days to apply the proper tension to the wound. On the 1st, 5th and 10th days after injury, 200 μl of PBS containing 100 μg of ADMSC-Exos was injected subcutaneously and intradermally with a 1 ml disposable syringe. Rats in the PBS group were injected with the same amount of PBS buffer solution, while rats in the control group were not subjected to any treatment. On days 0, 3, 7 and 14 after injury, the wounds were imaged with a camera (Canon, China) and measured with Image-Pro Plus 6.0 software. The rats were sacrificed 14 days after the operation for immunohistochemical analysis.

### Immunohistochemical Analysis

After fixation with formaldehyde, the skin samples excised from the wound sites were dehydrated in a low to high concentration alcohol gradient and immersed in xylene to obtain a transparent tissue mass. After paraffin embedding, the tissue blocks were cut into 4 µm sections. After the paraffin sections were dewaxed with xylene and rehydrated with anhydrous ethanol, antigen repair was performed with citric acid antigenic repair buffer (pH 6.0). Endogenous peroxidase activity was blocked with H_2_O_2,_ and the tissues were blocked with 3% BSA (Solarbio, China). After removing the blocking solution, the prepared primary antibodies against WNT2b (1:200; # ab203225; Abcam, UK), β-catenin (1:500; # ab32572; Abcam, UK), COL-I (1:100; # E-AB-62684; Elabscience, China), COL-III (1:100; # ab184993; Abcam, UK), and α-SMA (1:200; # ab5694; Abcam, UK) were added to the tissue sections, and the sections were incubated overnight at 4 °C. The tissue sections were washed with PBS (pH 7.4) 3 times for 5 min each. After the sections were dried slightly, the secondary antibody (HRP-conjugated) (Elabscience, China) of the species corresponding to that of the primary antibody was added to cover the tissues, and the tissues were incubated at room temperature for 50 min. The tissue sections were washed with PBS (pH 7.4) 3 times for 5 min each, and freshly prepared DAB (Solarbio, China) solution was added for colour development. The colour development time was controlled by observation under a microscope. After restaining nuclei with haematoxylin (Solarbio, China), the tissue sections were dehydrated and sealed. Micrographs were acquired using an FSX100 microscope (Olympus, Japan) for further analysis.

### Effects of WNT signaling pathway inhibitor (XAV939) on wound healing in rats

To verify that WNT/β-catenin pathway inhibitor can block the activation of ADMSC-Exos on WNT pathway in wound healing, a total of 9 male SD rats (270 ~ 290 g) were randomly divided into blank control group, XAV939 group, ADMSC-Exos treatment group and ADMSC-Exos + XAV939 (ADMSC-Exos + X) treatment group. The wound model was made in the same way as above. On day 1, day 5, and day 10 after injury, the ADMSC-Exos treatment group was injected with 100 μg exosome locally around the wound, and the ADMSC-Exos + XAV939 treatment group was injected with the same amount of exosome locally around the wound. XAV939 treatment group and ADMSC-Exos + X treatment group were intraperitoneally injected XAV939 (# M1796, AbMole, USA) four times on day 1, each time 1.25 mg/kg. XAV939 selectively inhibite downstream β-catenin in the WNT pathway. The control group was not treated. The wound healing of each group was recorded by camera on day 0, day 3, day 7, and day 14, and the wound area was calculated by Image-Pro Plus 6.0 software. The rats were sacrificed on the 14th day after surgery for H&E staining, Masson staining and immunohistochemical analysis.

### Isolation of Dermal Fibroblasts

Skin fibroblasts were isolated from male foreskin tissue. The outer epidermis and subcutaneous tissue were removed with sterile surgical scissors, and only the dermis was kept. The remaining white tissue was cut into square tissue blocks of approximately 0.5 × 0.5 cm^2^ in size and evenly spread on the bottom of a Petri dish (1 cm interval). An appropriate amount of complete medium supplemented with 15% FBS (Procell, China) was added to ensure that the medium completely covered the tissue blocks and that the bottom surface of each tissue block was close to the bottom of the Petri dish. The Petri dishes were transferred to a constant temperature incubator (SANYO, Japan) for culture at 37 °C in 5% CO_2_. Images of typical areas were acquired with a microscope (Olympus Corporation, Japan).

### Migration Assay

The effects of ADMSC-Exos on fibroblast migration were evaluated by a scratch assay. Briefly, fibroblasts were seeded in 6-well plates at 5 × 10^5^ cells/well and cultured in a humidified incubator containing 5% CO_2_ at 37 °C. When the cells were 90% confluent, the medium was replaced with fresh FBS-free medium after two washes with PBS, and the confluent cell monolayer was then scratched using a sterile 200 μl pipette tip. Different concentrations of ADMSC-Exos (0 μg/ml, 25 μg/ml, 50 μg/ml, and 100 μg/ml) were added to the wells. Images were acquired at 0 h, 12 h, and 24 h after the monolayer was scratched. The migration area was measured by using ImageJ software (Medical Cybernetics, USA) and quantified as follows: migration area (%) = (A^0^ – A^n^)/A^0^ × 100%, where A^0^ is the initial wound area (t = 0 h) and A^n^ is the residual area of the wound at the time of measurement (t = n h).

### Cell Growth Assay

Fibroblasts were seeded into 96-well plates at a density of 2 × 10^3^ cells/well for 6 h. The cells were then divided into 4 groups and treated with ADMSC-Exos in a concentration gradient of 0 μg/ml, 25 μg/ml, 50 μg/ml and 100 μg/ml. Each group had 6 replicate wells. 10 μl CCK-8 (MedChem Express, China) reagent was added into the 96-well plates at 12 h, 24 h and 48 h, respectively. The plates were incubated in an incubator at 37 °C for 2 h in the dark. The absorbance at 450 nm was measured with a microplate reader, and the proliferation of cells in each well was compared.

### Real-Time PCR Analysis

The expression of each gene was evaluated by qRT-PCR. Fibroblasts in logarithmic growth phase were inoculated into 6-well culture plates at 5 × 10^5^ cells/well. After the cells grew to a confluence of approximately 70%, the medium was removed by aspiration, and the cells were washed twice with PBS. Then, the cells were divided into 4 groups, which were treated with different concentrations of exosomes (0 μg/ml, 25 μg/ml, 50 μg/ml and 100 μg/ml) and cultured for 36 h for subsequent experiments.

RNA extraction from fibroblasts was performed using TRIzol® reagent (Takara, China) at 1 ml/well following the manufacturer’s instructions. Then, 300 ng of RNA was reverse transcribed into cDNA using a Prime Script RT Reagent Kit (Takara, China). Quantitative PCR was performed using an RT-PCR system (Takara, China) with SYBR Premix Ex Taq II (Takara, China) in a 10 μl reaction volume. After an initial denaturation step at 95 °C for 90 s, amplification was performed under the following thermal cycling conditions: 40 cycles of denaturation at 95 °C for 10 s, annealing at 55 °C for 10 s, and extension at 72 °C for 30 s. GAPDH was used as the reference gene for calculations. Expression levels were expressed relative to that of GAPDH via the comparative CT method. Oligonucleotides were synthesized by Integrated DNA Technologies (Takara, China). *COL-I* oligonucleotide primer sequences are as follows: forward, 5’-TAGGGTCTAGACATGTTCAGCTTTG-3’; reverse, 5’-CGTTCTGTACGCAGGTGATTG-3’. The sequences of *COL-III* are as follows: forward, 5’-TCAGGCCAGTGGAAATGTAAAGA-3’; reverse, 5’-CACAGCCTTGCGTGTTCGATA-3’. The sequences of *α-SMA* are as follows: forward, 5’- CTCTGGACGCACAACTGGCATC-3’; reverse, 5’-GGCATGGGGCAAGGCATAGC-3’.

### Western Blot Analysis

The exosome-specific protein markers CD9 and CD63 were analyzed by Western blotting. After protein sampling, electrophoresis and transfer to a PVDF membrane (Millipore, USA), protein bands were successfully obtained. The PVDF membrane was placed face up in an antibody incubator, and 3% skim milk powder (Yili, China) was added for blocking for 1.5 h. After blocking, the membrane was washed with TBST (Solarbio, China) 3 times for 10 min each. For primary antibody incubation, the PVDF membrane was immersed in a preprepared primary antibody solution (CD9 1:1500; CD63 1:1500) (# PA5-11,559, # 10628D; Invitrogen, USA) and placed in a refrigerator at 4 °C overnight. After primary antibody incubation, the membrane was washed with TBST 3 times for 10 min each, and the residual primary antibody solution was removed by washing. For secondary antibody incubation, the PVDF membrane was immersed in a goat anti-rabbit secondary antibody solution (1:1500) (Elabscience, China) prepared with 3% skim milk powder and incubated for 1.5 h. After secondary antibody incubation, the membrane was washed with TBST 3 times for 10 min each, and the residual secondary antibody solution was removed by washing. Finally, the PVDF membrane was developed and imaged, and the image analysis software ImageJ was used for analysis.

Western blotting assay was used to detect the expression of COL-I, COL-III, α-SMA and WNT2b/β-catenin in fibroblasts treated with ADMSC-Exos. Fibroblasts were seeded in a 6-well plate at a density of 5 × 10^6^ cells/well. After the cells grew to a confluence of approximately 70%, the cells were treated with different concentrations of exosomes (0 μg/ml, 25 μg/ml, 50 μg/ml and 100 μg/ml) and incubated at 37 °C in 5% CO_2_ for 36 h to detect collagen synthesis and the expression of α-SMA. As for the western blotting assay of WNT2b/β-catenin expression, the cells were treated with 2 ml of culture medium containing 50 μg/ml exosomes, PBS buffer or serum-free DMEM medium.

Then, the cells were treated with lysis buffer (RIPA protein lysis buffer: protease inhibitor = 100:1), and the concentration of the protein sample was determined with the BCA kit (Elabscience, China) according to the kit instructions. After adjusting the protein concentration, a 1/4 sample volume of loading buffer was added and mixed by vortexing. Proteins were denatured in a dry bath at 95 °C for 5 min. Primary antibodies against WNT2b (1:3000; # ab203225; Abcam, UK), β-catenin (1:5000; # ab32572; Abcam, UK), COL-I (1:2500; # E-AB-62684; Elabscience, China), COL-III (1:2500; # ab184993; Abcam, UK), α-SMA (1:2000; # ab5694; Abcam, UK), and GAPDH (1:2000; # E-AB-20079; Elabscience, China) were used. The corresponding goat anti-rabbit secondary antibody was also used. The remaining steps were the same as those used for Western blot analysis for the identification of exosome-specific protein markers. Signals were monitored with an Enhanced Chemiluminescence Detection System (Millipore, USA).

### Statistical Analysis

All experimental data were collected and analyzed with GraphPad 7. A t-test was used for comparisons between single groups, and one-way analysis of variance was used for comparisons among multiple (> 2) groups. The experimental data are expressed as the mean ± standard deviation (X ± SD) values. Differences were considered statistically significant when *P* < 0.05.

## Results

### Isolation and Characterization of ADMSCs

**Fig. 1:**
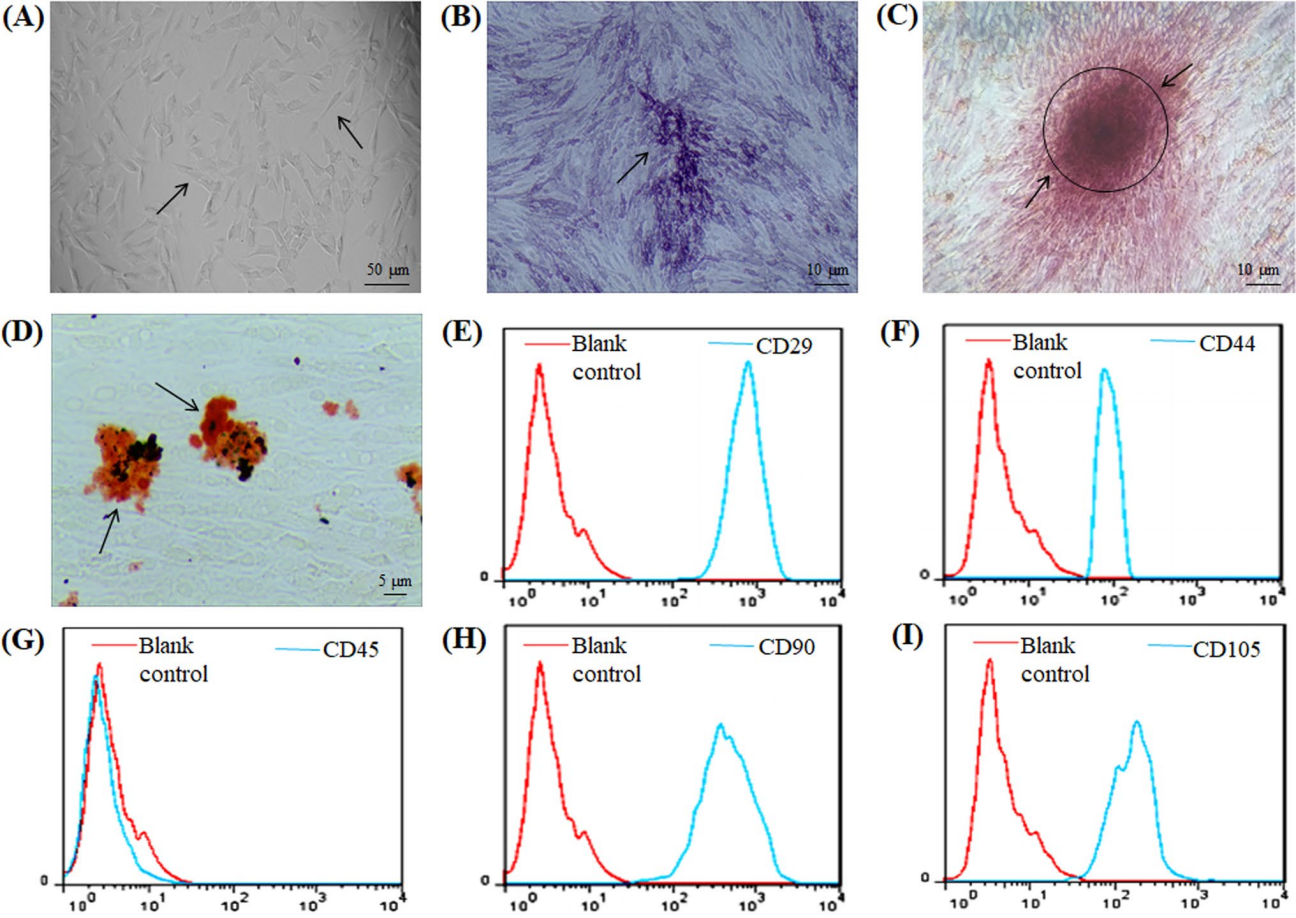
Characterization of ADMSCs. **A** Results of passage 3 ADMSCs observed under light microscope. **B** The alkaline phosphatase staining of ADMSCs on day 9 of osteogenesis is blue-purple, as shown by arrow. **C** On day 21 of osteogenic induction, the cells were lamellar with unclear structure, and red calcium nodules were observed in the extracellular matrix stained with Alizarin red S, as shown by arrow. **D** On day 14 of adipogenesis, fat vacuoles were stained orange by oil red O, as shown by the arrow. **E-I** Flow cytometry showed positive expression of CD29, CD44, CD90 and CD105, and negative expression of CD45

After initial isolation and culture for 36 h, ADMSCs were observed via a microscope, and most of the adherent cells were found to have a spindle‐like shape during cell culture. After the third passage, the morphology of ADMSCs gradually stabilized to an elongated spindle shape, similar to that of fibroblasts, with larger nuclei (Fig. 1A). Flow cytometric analysis of ADMSCs showed positive expression of the stem cell-specific markers CD29, CD44, CD90, and CD105 and negative expression of the haematopoietic marker CD45 (Fig. 1E-I). Osteogenic induction of ADMSCs showed bluish-purple alkaline phosphatase staining on the 9th day (Fig. 1B). On the 21st day of induction, the cells showed lamellar growth with an indistinct structure, and red calcium nodules were observed in the extracellular matrix by Alizarin red S staining (Fig. 1C). Adipogenic induction of ADMSCs showed that small vacuoles with good light transmissibility began to appear in the cytoplasm of adipogenic cells on the 4th day of adipogenic induction and that a large number of vacuoles of different sizes with good light transmissibility appeared on the 14th day of adipogenic induction. Oil red O staining appeared orange-red (Fig. 1D). The above results proved that ADMSCs were successfully isolated.

### Isolation and Characterization of ADMSC‐Exos

**Fig. 2:**
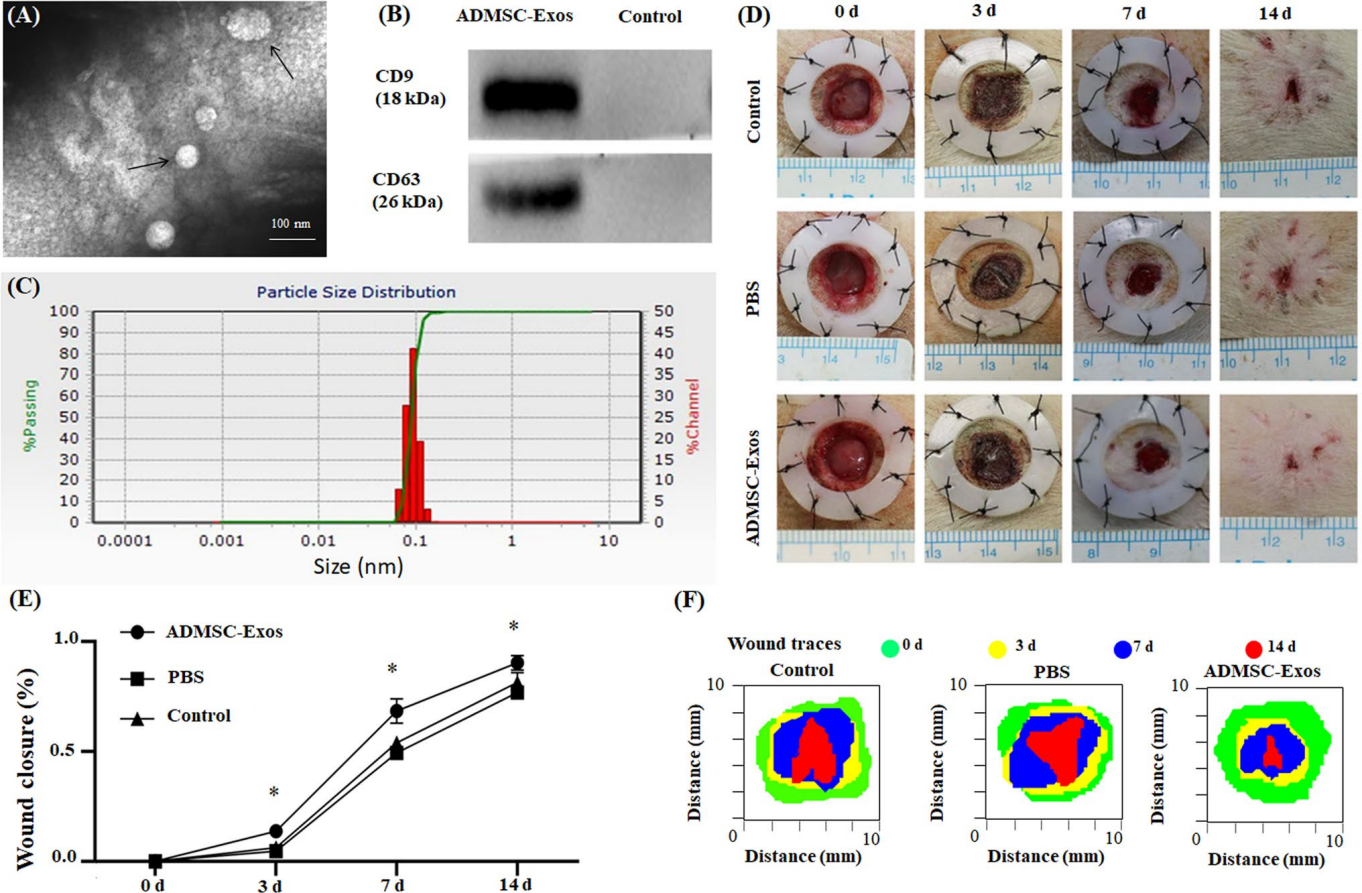
Exosomes were successfully isolated from ADMSCs medium; ADMSC-Exos promotes wound healing. **A** Exosomes were observed by transmission electron microscopy (TEM). **B** The expression of the exosome protein markers CD9 and CD63 was confirmed by Western blotting. **C** Nanoparticle tracking analysis (NTA) showed that the ADMSC-Exos distribution diameter ranged from 40 to 100 nm. **D**,** E** As can be seen from the wound images at different time points, the wound shrinkage rate of the AMDMSC-Exos group was significantly faster than that of the PBS group and the blank control group. At the 14th day, the remaining wound area was significantly reduced compared with the other two groups. **F** Traces of wound-bed closure during 14 days in vivo for each treatment category. The data were statistically analyzed using GraphPad. Results are presented as mean ± SD; *n* = 3; ^*^p < 0.05, compared with PBS and blank control group

The cup-shaped morphology of ADMSC‐Exos was observed by transmission electron microscopy (TEM) (100 000 × magnification) (Fig. 2A). Exosomes purified from ADMSC culture supernatants were characterized by particle size analysis, and the results showed that the exosomes were circular membrane-bound vesicles with a diameter of 40 to 100 nm (Fig. 2C), which was consistent with the previously reported exosome size distribution [25]. Western blotting also showed that the exosomal markers CD9 and CD63 were expressed in ADMSC‐Exos (Fig. 2B). These results indicated that exosomes from human ADMSCs were successfully isolated.

### ADMSC-Exos Accelerate Cutaneous Wound Healing in Rats

We locally injected 100 μg of ADMSC-Exos into full-thickness skin defects in rats at different time points (1, 5, 10 days) and photographed wound healing at different time points (0, 3, 7, 14 days). The wound healing rate was statistically analyzed using Image-Pro Plus 6.0, and the results showed that the wound healing rate in the ADMSC-Exos injection group was significantly faster than that in the PBS injection group or the blank control group, indicating the positive effect of ADMSC-Exos on wound healing (Fig. 2D, E, F).

### ADMSC-Exos can Promote Skin Fibroblast Proliferation and Migration

**Fig. 3:**
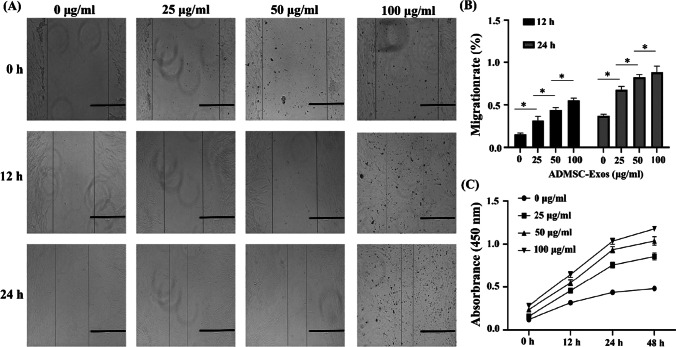
ADMSC-Exos promote fibroblasts cell proliferation and migration. **A**,** B** Representative images from the scratch wound assay and quantitative analysis of cell migration in each group at 12 h and 24 h. **C** CCK-8 analysis shows the proliferation results of fibroblasts at different concentrations of ADMSC-Exos. The data were statistically analyzed using GraphPad. Results are presented as mean ± SD; *n* = 3; ^*^p < 0.05, comparison between the two groups. Scale bars, 200 μm

To study the effect of ADMSC-Exos on the proliferation and migration of fibroblasts, we treated fibroblasts with different concentrations of exosomes (0 μg/ml, 25 μg/ml, 50 μg/ml, and 100 μg/ml), and the results of the CCK-8 assay showed that as the exosome concentration increased, the proliferation ability of fibroblasts was significantly increased compared with that of blank control cells (Fig. 3C) and that the differences in the proliferation ability among the groups treated with different concentrations were statistically significant (*P* < 0.05). In addition, the scratch assay results showed that the migration rate of fibroblasts increased with increasing exosome concentration (Fig. 3A, B) and that the differences in the migration rate among the groups treated with different concentrations were statistically significant (*P* < 0.05). ADMSC-Exos showed concentration-dependent effects on the proliferation and migration of fibroblasts; 100 μg/ml ADMSC-Exos had the most significant effect. These results suggest that ADMSC-Exos can promote the proliferation and migration of fibroblasts.

### ADMSC-Exos Promoted Collagen Synthesis and Decreased α-SMA Synthesis in Fibroblasts

**Fig. 4:**
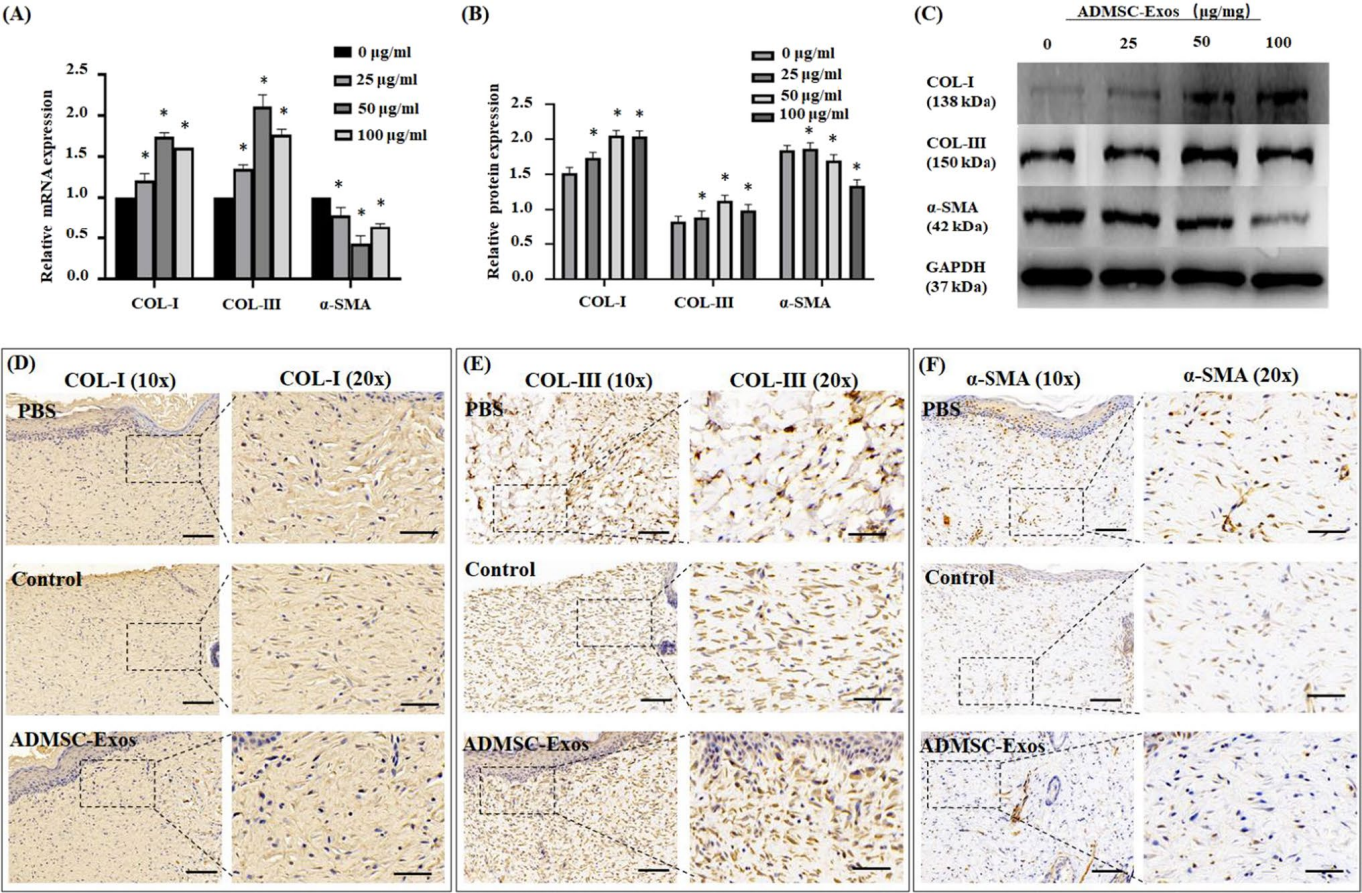
In vitro and in vivo, ADMSC-Exos affects the synthesis of collagen and α-SMA in fibroblasts. **A** The qPCR analysis of the mRNA expression of *COL-I*, *COL-III*, *α-SMA* in human fibroblasts treated with different concentrations of ADMSC-Exos. **B, C** Western blotting of the protein expression level of COL-I, COL-III, α-SMA in fibroblasts treated with different concentrations of ADMSC-Exos. **D, E, F** Representative images of immunohistochemistry showed COL-I, COL-III, α-SMA expression in the designated treatment group. The data were statistically analyzed using GraphPad. Results are presented as mean ± SD; *n* = 3; ^*^p < 0.05, compared with 0 μg/ml group. Scale bars, 100 μm (10x), 50 μm (20x)

The mRNA and protein expression levels of COL-I, COL-III and α-SMA in fibroblasts treated with ADMSC-Exos were analyzed by Western blotting and qRT-PCR. The results showed that ADMSC-Exos could promote collagen synthesis by stimulating the synthesis of COL-I and COL-III in fibroblasts and that this promotive effect was enhanced as the concentration of ADMSC-Exos increased. At the same time, the expression of α-SMA was decreased, indicating that myofibroblast differentiation was inhibited by ADMSC-Exos (Fig. 4A, B, C). Interestingly, at the high concentration of ADMSC-Exos (100 μg/ml), the collagen synthesis ability of fibroblasts was decreased compared with that at the low concentration, but it was still higher than that at 0 μg/ml. Immunohistochemical staining of rat wounds also showed that the expression of COL-I and COL-III was increased and that of α-SMA was decreased in the ADMSC-Exos group (Fig. 4D, E, F).

### ADMSC-Exos may Promote Wound Healing by Activating WNT/β-catenin Signaling

**Fig. 5:**
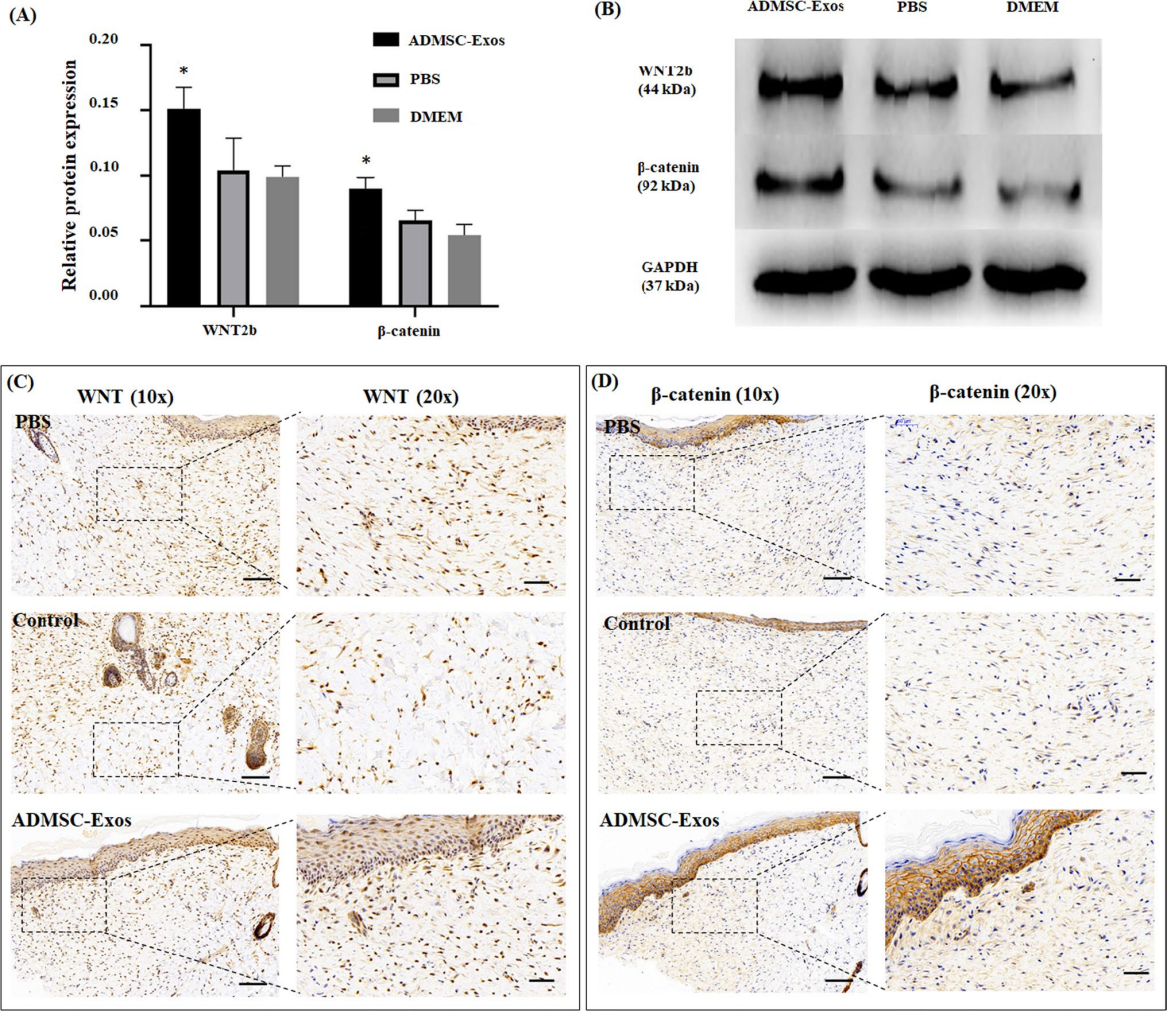
ADMSC-Exos may promote wound healing via activating the WNT/β-catenin signaling pathway. **A**,** B** Western blotting of the protein expression level of WNT2b and β-catenin in fibroblasts treated with 50 μg/ml ADMSC-Exos、PBS and non-FBS DMEM for 36 h. **C**,** D** Representative images of immunohistochemistry showed WNT2b and β-catenin expression in the designated treatment group. The data were statistically analyzed using GraphPad. Results are presented as mean ± SD; *n* = 3; ^*^p < 0.05, compared with PBS and DMEM group. Scale bars, 100 μm (10 x), 50 μm (20 x)

To investigate the potential molecular mechanism of ADMSC‐Exos in wound healing, we performed two experimental protocols in vivo and in vitro. First, ADMSC-Exos were applied to normal skin fibroblasts, and the protein expression levels of WNT2b and β-catenin were determined by Western blotting. Second, we performed immunohistochemical analysis to detect the protein expression of WNT2b and β-catenin in the scar tissue of rats after treatment with ADMSC-Exos. We found that in both the in vivo and in vitro experiments, the expression of WNT2b and β‐catenin was noticeably enhanced in the ADMSC-Exos group compared with the control group (Fig. 5A-D), suggesting that ADMSC-Exos may promote wound healing by activating the WNT/β-catenin signaling pathway.

### Activation of WNT Pathway by ADMSC-Exos was Inhibited by XAV939

**Fig. 6:**
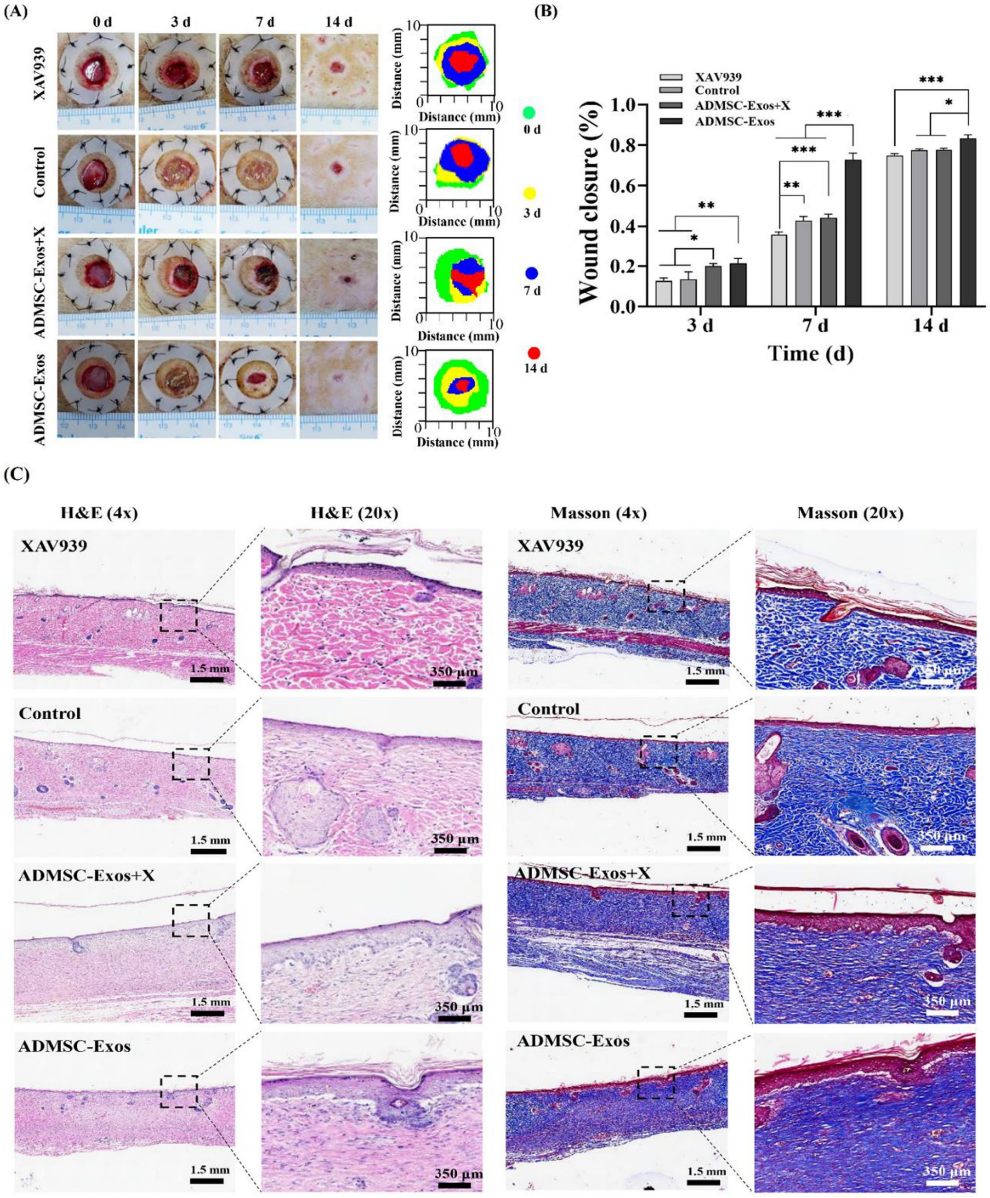
Effect of ADMSC-Exos on wound healing after application of WNT2b/β-catenin pathway inhibitor. **A**,** B** Digital photographs of wound area of the control group, ADMSC-Exos group and ADMSC-Exos + X group at 0, 3, 7, 14 days after operation. Histograms showed the relative wound closure rates analyzed by Image-Pro Plus 6.0 software. **C** Representative H&E and Masson trichromatic staining images of wound tissue sections of the control group, ADMSC-Exos group or ADMSC-Exos + X group on day 14 after operation, scale bar = 1.5 mm, 350 μm. The data was shown as mean ± SD (*p < 0.05, **p < 0.01, ***p < 0.001; *n* = 3)

We applied WNT2b/β-catenin pathway inhibitor (XAV939) to further observe the changes of ADMSC-Exos on wound healing. After treatment with WNT pathway inhibitor, the wound closure rate of ADMSC-Exos + X treatment group decreased compared with that of ADMSC-Exos treatment group, and the difference was statistically significant on day 7 and 14, suggesting that blocking the WNT2b/β-catenin pathway can inhibit the wound healing promoted by ADMSC-Exos (Fig. 6A, B). Although there was no statistical difference in the healing rate between the XAV939 treatment group and the blank control group on day 3 and 14, the healing rate in the XAV939 treatment group was significantly lower than that in the blank control group on day 7, suggesting that the activation of the WNT pathway has a positive effect on wound healing. H&E analysis showed that there was no obvious inflammatory cell infiltration in the three groups on the 14th day, and the fibrous tissue became dense, orderly and continuous with epithelialization, especially in the ADMSC-Exos group, followed by the ADMSC-Exos + X group. Masson analysis showed that ADMSC-Exos group had more compact collagen and the most orderly and continuous arrangement. In the XAV939 treatment group, the collagen arrangement was disordered, coarser and sparse and the degree of the upper cortex was minimal (Fig. 6C ).

**Fig. 7:**
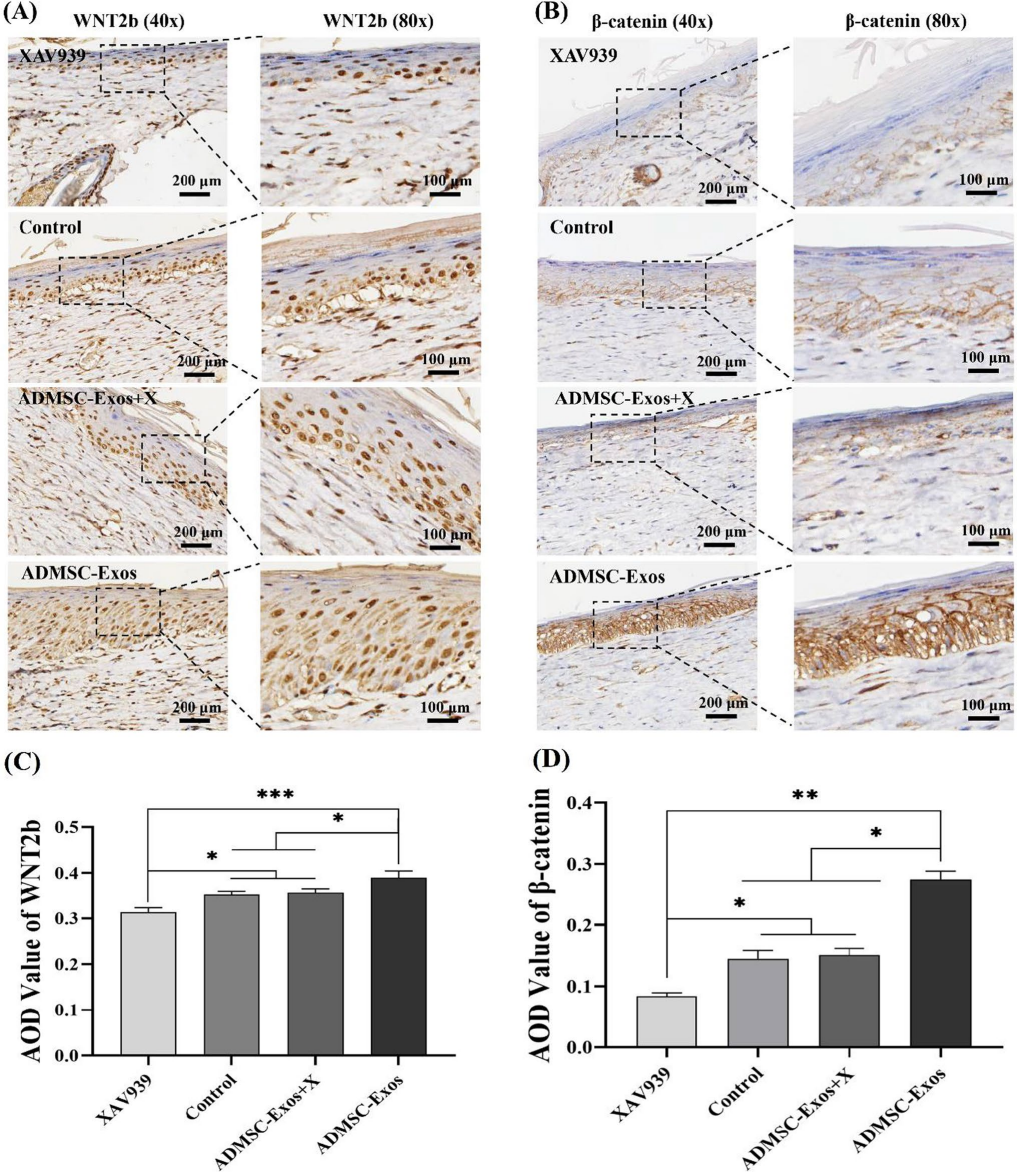
Immunohistochemical analysis after use of WNT pathway inhibitor (XAV939). WNT2b (**A**,** C**) and β-catenin (**B**,** D**) detected in wounds on the 14th day after use of WNT pathway inhibitor by immunohistochemical staining, Scale bar = 100 µm,200 μm. The data are the mean ± SD (*n* = 3; *p < 0.05, **p < 0.01)

After XAV939 treatment, immunohistochemical average optical density (AOD) analysis showed that the expression of WNT2b protein in the ADMSC-Exos increased, which was higher than that in the ADMSC-Exos + X treatment groups, control group, and XAV939 treatment group, while XAV939 treatment group had the lowest expression (Fig. 7A, C); β-catenin in ADMSC-Exos group was higher than that in ADMSC-Exos + X treatment group, XAV939 treatment group and control group (Fig. 7B, D). The expression level of β -catenin in XAV939 treatment group was the lowest. These results showed that XAV939 blocked the activation of ADMSC-Exos on WNT pathway during wound healing.

## Discussion

Wound healing is one of the most complex biological processes, requiring the coordinated cooperation of multiple cells and the accurate coordination of various biological and molecular events [26]. Sufficient studies have demonstrated that stem cells play an important role in tissue repair [10–12], but limitations, such as ethics, immune rejection, and poor control, have hindered their use. Exosomes have a significant effect on intercellular communication [27], which have been reported to be ideal candidates for tissue repair and are emerging as a promising alternative to cell-based therapies [28], so the basic research and translational application of exosomes in regenerative medicine have become a research focus [29–32]. Unfortunately, the snag is that, because of the complex composition of exosomes, little is known about their mechanisms [32]. Therefore, in this study, we focused on the role of ADMSC-Exos in the process of wound healing and discussed the relationship between ADMSC-Exos and WNT/β-catenin signaling pathway.

Ultracentrifugation (UC) is widely used for exosome isolation due to the high achievable purity and low cost [33]. So in this study, we applied UC to isolate ADMSC-Exos. Particle size analysis(Fig. 2C), TEM photography (Fig. 2A) and exosome specific antibody expression (Fig. 2B) were consistent with the results of other previous studies on exosomes [34, 35], which proved that we successfully obtained exosomes through UC and UC is a reliable way to obtain exosomes. Karlien et al. found that local injection of ADMSCs could preserve drugs with high wound closure rate, which was superior to caudal vein injection [36]. Therefore, we used the local injection method in our study.

It is well established that dermal fibroblasts, the primary producers and organizers of the ECM needed to restore tissue integrity after injury, are a key component of wound healing, whose functional status determine the speed of repair and the size of scar after healing [37]. Hyperactivation and prolonged action of fibroblasts lead to tissue contractures that ultimately hinder organ function. Conversely, insufficient fibroblast activation and activities interfere with normal wound healing. In this study, by assessing the effect of ADMSC-Exos on fibroblasts of human in vitro, we found that ADMSC-Exos promoted the proliferation and migration of fibroblasts and upregulated the expression of COL-I and COL-III, which was undoubtedly helpful to accelerate wound healing. Interestingly, at a concentration of 100 μg/ml, the ability of ADMSC-Exos to enhance collagen synthesis in fibroblasts was weakened, while fibroblast proliferation and migration did not decline. Meanwhile, by evaluating the effect of ADMSC-Exos on wound healing of rats, we found that ADMSC-Exos made collagen arrangement more orderly and regular. As everyone knows, collagen (COL-I, COL-III), a wound-filling component secreted by fibroblasts and myofibroblasts during wound healing [38], is the most abundant component in developing ECM [39]; collagen deposition early in wound healing contributes to wound healing, but later collagen deposition leads to the formation of unsightly scars and organ dysfunction [40]. As for our results, the high concentration of ADMSC-Exos reduced collagen formation and increased the migration and proliferation of fibroblasts, which may be one of the reasons why ADMSC-Exos accelerated wound healing and weakened scar formation. Unfortunately, the specific mechanism is still unclear and needs further study. We hypothesized that these effects were controlled by the complex contents of exosomes, which was shown by our concentration gradient assay.

For another, α-SMA is the marker of fibroblast differentiation into myofibroblast [41], which is closely related to the mechanical tension of wound surface, and excessive α-SMA expression is also the cause of promoting scar formation. Regarding the decreased expression of α-SMA in our research, we predicted that the application of ADMSC-Exos to the wound increased the migration rate of fibroblasts from the periphery to the centre of the wound surface, thus reducing the surface tension. Meanwhile, we speculated that ADMSC-Exos may have been able to inhibit the conversion of fibroblasts to myofibroblasts, thereby inhibiting excessive scar formation.

The WNT/β-catenin signaling pathway serves a vital role in proliferation, differentiation, movement, and morphology of cells, and it is also involved in mediating stem cell pluripotency [20]. β‐Catenin, a subunit of the cadherin protein complex, is an integral component of the classic WNT signaling pathway [42]. Accumulating evidence has confirmed that activation of the WNT/β‐catenin signaling pathway plays an essential role in the proliferative phase of wound healing [42, 43]. Our previous studies showed that WNT-responsive oral mucosal stem cells are activated in response to oral mucosal injury, thereby accelerating wound healing [21]. Thus, according to the conclusion that ADMSC-Exos activated WNT/β-catenin signaling pathway in this study, we have reason to deduce that whether ADMSC-Exos can also promote skin stem cell differentiation through the WNT signaling pathway during wound healing remains to be further studied. Moreover, our previous studies also showed that WNT-responsive stem cells can regulate periodontal membrane fibrosis and alveolar bone density to adapt to changes in mechanical stress [22]. Therefore, it remains to be further studied whether the effect of ADMSC-Exos on skin wound healing can also regulate the effect of mechanical tension by regulating WNT-responsive stem cells, which would be significant. At present, exosomes are an emerging platform and a critical factor in facilitating WNT secretion and transport. Zhang et al. found that exosomes from human umbilical cord MSCs can deliver WNT4 to target cells and activate β-catenin nuclear translocation, which has a positive effect on wound healing [44]. Ma et al. found that ADMSC-Exos could increase the protein expression of WNT and β-catenin in HaCaT cells treated with hydrogen peroxide to simulate skin damage[35]. However, their study did not research the effect of exosomes on fibroblasts during wound healing, and did not investigate the expression of ECM. Our study just complemented this and further refined the mechanism of exosomes through WNT signaling pathway. In this study, the elevated expression of β‐catenin in vitro and in vivo under treatment with ADMSC-Exos suggested that WNT/β‐catenin signaling may be related to the underlying mechanism of ADMSC‐Exos in wound healing. However, because of the complexity of wound tissue healing, more studies should be carried out on the association between the WNT/β-catenin signaling pathway and cutaneous wound healing.

## Conclusion

In summary, ADMSC-Exos implies a pivotally stimulative role for wound healing process, in which this study revealed that WNT/β-catenin signaling pathway was involved. Furthermore, ADMSC-Exos had a preventive effect on cicatrization by boosting the proliferation and migration of fibroblasts, downregulating the expression of fibroblast α-SMA, and promoting the rearrangement of collagen fibres concurrently, which showed great possibilities for wound applications, providing novel treatment strategies for wound healing.

## Data Availability

The data underlying this article will be shared on reasonable request to the corresponding author.
